# The Ultraviolet Irradiation of Keratinocytes Induces Ectopic Expression of LINE-1 Retrotransposon Machinery and Leads to Cellular Senescence

**DOI:** 10.3390/biomedicines11113017

**Published:** 2023-11-10

**Authors:** Fadi Touma, Marine Lambert, Amelia Martínez Villarreal, Jennifer Gantchev, Brandon Ramchatesingh, Ivan V. Litvinov

**Affiliations:** 1 Research Institute, McGill University Health Centre, McGill University, Montreal, QC H4A 3J1, Canada; fadi.touma@mail.mcgill.ca (F.T.); brandon.ramchatesingh@mail.mcgill.ca (B.R.); 2 Faculty of Medicine and Health Sciences, McGill University, Montreal, QC H3G 2M1, Canada; 3 Department of Dermatology, McGill University, Montreal, QC H4A 3J1, Canada

**Keywords:** long interspersed nucleotide element-1 (LINE-1), phototherapy, keratinocytes, BB-UVB, NB-UVB, genomic instability, cellular senescence, microRNAs, *miR-16*, HaCaT, N/TERT1

## Abstract

Retrotransposons have played an important role in evolution through their transposable activity. The largest and the only currently active human group of mobile DNAs are the *LINE-1* retrotransposons. The ectopic expression of *LINE-1* has been correlated with genomic instability. Narrow-band ultraviolet B (NB-UVB) and broad-band ultraviolet B (BB-UVB) phototherapy is commonly used for the treatment of dermatological diseases. UVB exposure is carcinogenic and can lead, in keratinocytes, to genomic instability. We hypothesize that *LINE-1* reactivation occurs at a high rate in response to UVB exposure on the skin, which significantly contributes to genomic instability and DNA damage leading to cellular senescence and photoaging. Immortalized N/TERT1 and HaCaT human keratinocyte cell lines were irradiated in vitro with either NB-UVB or BB-UVB. Using immunofluorescence and Western blotting, we confirmed UVB-induced protein expression of LINE-1. Using RT-qPCR, we measured the mRNA expression of *LINE-1* and senescence markers that were upregulated after several NB-UVB exposures. Selected miRNAs that are known to bind *LINE-1* mRNA were measured using RT-qPCR, and the expression of *miR-16* was downregulated with UVB exposure. Our findings demonstrate that UVB irradiation induces *LINE-1* reactivation and DNA damage in normal keratinocytes along with the associated upregulation of cellular senescence markers and change in *miR-16* expression.

## 1. Introduction

Phototherapy using different forms of ultraviolet (UV) light is routinely used to treat numerous dermatologic conditions such as atopic dermatitis, psoriasis, and vitiligo. UV light covers the wavelengths 100–400 nm and can be classified, depending on the wavelength range, into UVA (315–400 nm), UVB-broad band (BB-UVB) (280–315 nm), UVB-narrow band (NB-UBV) (311–313 nm), and UVC (100–280 nm) [1,2,3]. While UV radiation below 290 nm is almost imperceptible at ground level due to the rare presence of stratospheric ozone, which absorbs the majority of radiation with wavelengths below 310 nm, it is essential to note that a portion of UVB radiation, which does reach us, can still be detrimental to unprotected living cells, primarily through DNA and protein damage [2,4,5]. UV radiation, particularly UVB, plays a pivotal role in regulating diverse homeostatic activities within the body. It acts as a trigger that influences not only the skin’s defense mechanisms but also its ability to restore cutaneous homeostasis, impacting the central nervous, endocrine, and immune systems in a highly coordinated manner [6]. In addition to initiating mechanisms related to maintaining skin integrity and overall homeostasis within the body, the absorption of UV radiation by the skin can lead to skin-related pathologies such as cancer, aging, and autoimmune reactions. It has been shown that UVB irradiation plays a role in autoimmunity through the induction of autoantibody production following the antigenic presentation of UV-stimulated viral and cellular proteins. The promoter-driven transcription of human endogenous retroviral sequences and the expression of those sequences, which are aberrantly activated in autoimmune diseases such as lupus erythematosus, were induced following the treatment of primary epidermal keratinocytes or HaCaT cells with UVB [7]. The current evidence suggests that UV treatments using UVA (especially in the presence of a psoralen) or BB-UVB induce cutaneous carcinogenesis resembling or at times exceeding sunlight exposure [8,9,10]. Phototherapy using UV is recognized as a carcinogen contributing to the development of basal cell carcinoma (BCC), cutaneous squamous cell carcinoma (cSCC), melanoma, and Merkel cell carcinoma [11,12,13,14,15,16]. While UVA irradiation affects the deeper reticular dermal layers, UVB irradiation primarily affects the epidermis and papillary dermis [17,18]. UV irradiation of the skin leads to the generation of reactive oxygen species, direct DNA damage, induction of the p53 response, and inhibition of DNA synthesis. UVB-induced genomic instability occurs through a photochemical reaction which leads to the dimerization of cytosine and thymine pyrimidine bases and the formation of UV products, most commonly, cyclobutane pyrimidine dimers (CPDs) and pyrimidine (6-4) pyrimidone photoproducts (64PPs) [18,19,20,21,22]. These DNA photoproducts block transcription and are mutagenic, leading to skin cancer development [18,19,20,21,22]. However, recent evidence suggests that additional UV-induced mechanisms of genomic instability may be at play. Interestingly, solar and artificial UV exposure have been shown to cause reactivation of retrotransposons in the skin, contributing to genomic instability and carcinogenesis [23]. Nonetheless, the regulation and downstream effects of UV-induced retrotransposition in normal/non-transformed human keratinocytes remains poorly understood. If further investigated, retrotransposition can better our understanding of UV-induced cellular damage and help to mitigate UV-associated dermatologic diseases, including cellular senescence and photoaging.

Transposable elements, which played an important role in the evolution of the human genome, are DNA sequences that can move from one site to another within the genome and are further classified into transposons and retrotransposons [24,25,26]. While transposons excise and paste themselves from and into the genome, retrotransposons use RNA intermediates that are reverse-transcribed to be inserted into new sites within the genome [25]. Retrotransposons, which comprise ~40% of the mammalian genome, are also subdivided into two groups, i.e., long terminal repeat (LTR) retrotransposons and non-LTR retrotransposons, whereas human LTR elements are endogenous retroviruses. While transposons are inactive and LTR retrotransposons’ activity is very limited in the human genome, non-LTR retrotransposons are the only currently active elements in the human genome [27,28,29,30,31]. Non-LTR retrotransposons include long interspersed nuclear element (LINE)-1, *Alu* and *SVA* elements that comprise together about one-third of the human genome [32]. *LINE-1* represents the largest, and the only autonomously active transposable element in the human genome. *LINE-1* elements constitute 17% of the human genome and are ~6 kilobases in length [32]. *LINE-1* consists of 5′ and 3′ untranslated regions (UTRs) containing an internal polymerase II bidirectional promoter and polyadenylation signal, respectively, and two open reading frames (ORF1 and ORF2) in between that encode ORF1p and ORF2p [33,34]. ORF1p (~40 kD) is an RNA-binding protein with chaperone activity, and ORF2p (~150 kD) has endonuclease and reverse-transcriptase activities. Together they enable a target-primed reverse transcription process of retrotransposition to insert their sequences into the genome [29,34,35,36,37,38]. A variety of genetic alterations such as gene insertions, deletions, and inversions have been shown to be induced by *LINE-1* retrotransposons [39,40].

The molecular mechanism behind retrotransposon reactivation and downstream regulation remains poorly understood. The aberrant reactivation of *LINE-1* in response to cellular stressors such as UV exposure or methylation changes in the cell has been linked to deleterious effects leading to DNA damage, cellular senescence, and photoaging [41,42]. There is only a limited set of regulatory RNAs and proteins that have been suggested to be linked to their regulation in model organisms of germ or somatic cells during different stages of ontogenesis. Examples of regulatory agents include P-element-induced wimpy testis (PIWI)-interacting RNAs (piRNAs), DNA methyltransferase 3 (DNMT3), germ cell tumor-specific factor 1 (GTSF1), and heat shock proteins [34,35,43,44,45,46]. The importance of miRNA signaling in human diseases has been documented, and many miRNA-based treatments have been developed such as the use of cobomarsen in dermatology to target *miR-155* to treat skin lymphomas [47,48,49]. Although piRNAs have been shown to play an important role in *LINE-1* regulation, their expression alone does not fully explain the dynamics of *LINE-1* expression [50]. The expression of the purified *U1 noncoding RNA* (*RNU1*) has also been indicated to serve as an important sensor of UV damage [51]. However, this signaling event has not been directly linked to retrotransposon reactivation. Therefore, other small non-coding RNAs (sncRNAs) that are yet to be discovered may play an important role in *LINE-1* reactivation.

## 2. Materials and Methods

### 2.1. Cell Cultures and Treatment Conditions

This study used two types of human keratinocyte cell lines: N/TERT1 cells (neonatal male) obtained from the laboratory of Dr. James Rhinewald at Harvard University [52,53] and HaCaT cells (62-year-old adult male) provided by Dr. Anie Philip’s lab at McGill University [54]. The HaCaT cells were grown in Dulbecco’s Modified Eagle’s Medium (DMEM) (ATCC Cat# 30-2002, Manassas, VA, USA), while the N/TERT1 cells were grown in Defined Keratinocyte Serum-Free Medium (Thermo Fisher Scientific Gibco Cat# 10744019, Waltham, MA, USA). For UV irradiation, after 24 h of incubation, cells were washed twice with phosphate-buffered saline (PBS) without Mg^2+^ and Ca^2+^, pH 7.5, and were irradiated at 50–70% confluency. Cells were treated with 15 mJ/cm^2^ of BB-UVB or 75 mJ/cm^2^ of NB-UVB radiations based on pilot experiments. Using a UVB handheld lamp (Dermfix 1000MX, Augsburg, Germany), BB-UVB irradiation was delivered with a fluorescent bulb emitting 280–315 nm wavelengths with a peak at 313 nm (Philips PL-S9W/12/2P twin bulb, Piła, Poland), whereas NB-UVB was delivered using a fluorescent bulb emitting a primary wavelength of 311 nm (Dermfix 9W UVB Narrowband twin bulb, Augsburg, Germany). The cells were subjected to one or more irradiations with either NB-UVB (75 mJ/cm^2^) or BB-UVB (15 mJ/cm^2^) in tissue culture-treated 6-well plates under 37 °C and 5% CO_2_.

### 2.2. Immunofluorescence

Immunofluorescence assays were performed in 4-well-chambered cell culture slides (Corning Falcon #354114, New York, NY, USA) following one or more UV irradiations at a 1-hour time point. The antibodies used included recombinant rabbit monoclonal antibody against *LINE-1* ORF-1p (Abcam Cat# ab245249 (clone #EPR22227-6), Waltham, MA, USA, RRID: AB_2941773), 1:100 dilution in 1% BSA in PBS, and rabbit polyclonal antibody to γH2AX (phospho S139) (Abcam Cat# ab11174, Waltham, MA, USA, RRID: AB_297813), 1:1000 dilution in 1% BSA in PBS, and appropriate secondary antibodies, as detailed before [55], counterstained with 4′,6-diamidino-2-phenylindole (DAPI) nuclear marker (Thermo Fisher Scientific Invitrogen #D1306, Waltham, MA, USA, RRID: AB_2629482). Nuclear size and cell diameter measurements were obtained from individual counts of 50–70 N/TERT1 cells per UVB condition following 6 repeated UV irradiations with either BB-UVB or NB-UVB (24 h after treatment). Imaging was performed using an Etaluma Lumascope LS720 (Etaluma, Carlsbad, CA, USA) with a 60X objective (Meiji Techno, MA969, Campbell, CA, USA) and quantified using the immunofluorescence protocol of the QuPath open-source software (v0.3.2) [56].

### 2.3. Western Blotting

Protein extracts were obtained using the RIPA Lysis and Extraction Buffer (Thermo Fisher Scientific Cat# 89900, Waltham, MA, USA) from N/TERT1 cells after 6 repeated UV irradiations with either NB-UVB or BB-UVB 24 h after exposure. Western blotting was performed to measure the expression of ORF1p using a recombinant mouse monoclonal anti-*LINE-1* ORF-1p antibody (Sigma-Aldrich Cat# 9Q01FP (clone #4H1), Millipore, Oakville, ON, Canada, RRID: AB_2941775), 1:1000 dilution in 1% BSA in PBS, with a loading control of a polyclonal anti GAPDH antibody (Thermo Fisher Scientific Cat# PA1-987, Waltham, MA, USA, RRID: AB_2107311), 1:2000 dilution in 1% BSA in PBS, and Clarity™ Western ECL Substrate (BioRad Cat# 1705060, Hercules, CA, USA).

### 2.4. RT-qPCR

For the mRNA analysis, 100,000 HaCaT or 300,000 N/TERT1 cells were lysed using QIAzol Lysis Reagent (Qiagen Cat# 79306, Germantown, MD, USA) following one or more UV exposures at different time points (1, 3, 6, and 24 h; after the last irradiation). Total RNA was extracted using an miRNeasy kit (Qiagen Cat# 217084). The extracted RNA was verified for quantity and yield using spectrophotometry (BioDrop DUO+ MBI Cat# 80-3006-68, Montreal, QC, Canada). RNA (1000 ng) was retrotranscribed to synthesize cDNA with an iScript™ Advanced cDNA Synthesis Kit (BioRad Cat# 1725037) for subsequent use in qPCR. qPCR was performed using SsoAdvanced Universal SYBR^®^ Green Supermix (BioRad Cat# 1725274) to measure the relative mRNA expression of ORF2 of *LINE-1*, as well as senescence markers IFN-B, IL-6, IL-8, MMP1, and MMP3 to the β-actin housekeeping mRNA expression. The sequence of primers used in the RT-PCR is detailed in Table A1 (Thermo Fisher Scientific Invitrogen™ Cat# 10336022, Waltham, MA, USA). For the miRNA analysis, 300,000 N/TERT1 cells were lysed using QIAzol Lysis Reagent (Qiagen Cat# 79306) following repeated UV exposures at different time points (1, 3, 6, and 24 h; after the last irradiation). Total RNA was extracted, and miRNA-enriched fractions were separated from larger RNAs using an miRNeasy kit (Qiagen #217084). The extracted RNA was verified for quantity and yield using spectrophotometry (BioDrop DUO+ MBI Cat# 80-3006-68). miRNA-enriched RNA (50 ng) was retrotranscribed to synthesize cDNA using an miRCURY LNA RT Kit (Qiagen Cat# 339340) for subsequent use in qPCR. qPCR was performed using an miRCURY LNA miRNA PCR Assay (Qiagen Cat# 339306) to measure the relative miRNA expression of *RNU1A1, hsa-miR-16-5p, hsa-miR-125b-1-3p, hsa-miR-138-5p, hsa-miR-22-5p, hsa-miR-197-3p, hsa-miR-487b-5p*, and *hsa-let-7a-5p*, to the *U6 snRNA* expression. The GeneGlobe IDs are provided in Table A2.

### 2.5. Statistical Analysis

Ordinary one-way, mixed-effect model or Kruskal–Wallis nonparametric analysis was performed and corrected for multiple comparisons using Dunnett’s or Tukey’s test using GraphPad Prism version 10.0.2 for Mac, GraphPad Software, Boston, MA, USA, www.graphpad.com (accessed on 30 July 2023) (RRID: SCR_002798). The statistical analysis and correction tests were chosen with the help of the software depending on the type of generated data. Significance was defined as *p* < 0.05, and all experiments were performed in triplicates, with error bars representing the mean ± S.E.M.

## 3. Results

### 3.1. UV Irradiation Is Associated with DNA Damage and Impaired Proliferation

**Figure 1:**
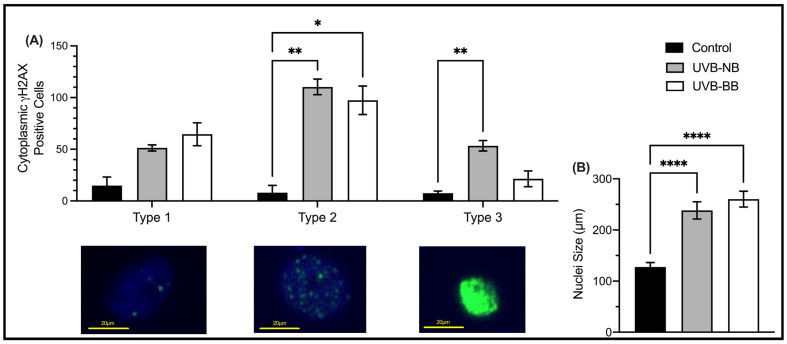
UV irradiation induces DNA damage. (**A**) The expression of γH2AX (green) in N/TERT1 keratinocytes as shown by immunofluorescence staining following 24 h of UV irradiation with either NB-UVB or BB-UVB as compared to unirradiated control samples (*n* = 3 with 500 cells/condition). The three patterns of γH2AX staining correspond to the number of double-stranded DNA breaks, i.e., type 1 expression <10 nuclear foci (low-level DNA damage), type 2 expression >10 nuclear foci (high-level DNA damage), and type 3 pan-nuclear expression (pre-apoptotic state). The photos were taken on an Etaluma Lumascope LS720 microscope with a 60X objective (Meiji MA969) (scale bar 10 μm). Significance was calculated using a mixed-effects model to analyze the data, and for multiple comparisons correction, Dunnett’s test was applied. (**B**) Nuclear size counts of NTERT cells after 6 UV irradiations (*n* = 3 with 50–69 cells/condition). Significance was calculated using the one-way ANOVA test and was corrected for multiple comparisons using Dunnett’s test. (**** *p* value < 0.0001, ** *p* value < 0.0021, * *p* value < 0.05).

To quantify the impact of UV irradiation on the DNA of normal keratinocytes, we performed immunofluorescence for the DNA damage marker γH2AX in irradiated cells. The double-stranded DNA breaks (DSBs) marker γH2AX [57] was shown to be upregulated in N/TERT1 cells upon BB-UVB and NB-UVB repeated irradiation (Figure 1A). Three patterns of immunofluorescence staining for γH2AX were identified [55]. These patterns are correlated with the levels of DNA damage, where type 1 corresponds to low level of DNA damage (<10 γH2AX nuclear foci), type 2 corresponds to high level of DNA damage (>10 nuclear foci), and type 3 is indicative of pan-nuclear staining which is associated with a pre-apoptotic phenotype [58,59,60,61,62]. The extent of DNA damage varied, as indicated by the recruitment of γH2AX, according to the type of UV irradiation used. Both BB-UVB and NB-UVB irradiations resulted in increase in types 1–2 γH2AX staining.

**Figure 2:**
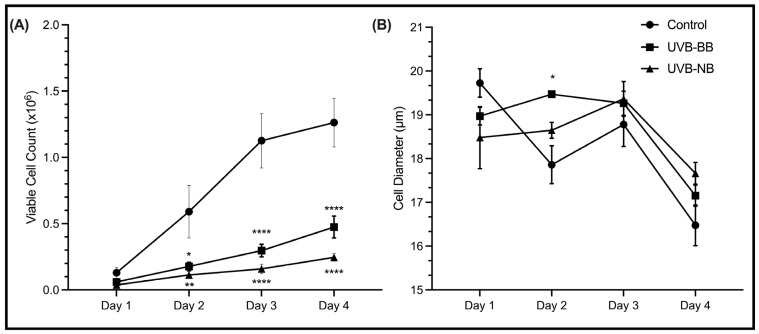
UV irradiation decreases cell proliferation and increases cell diameter. (**A**) The proliferation curve of HaCaT (*n* = 3) cells at 1, 2, 3, and 4 days following 6 UV irradiations with either NB-UVB or BB-UVB as compared to unirradiated control samples. (**B**) Cell diameter of HaCaT (*n* = 3) cells after 6 UV irradiations (50–70 cells/condition). The photos were taken on an Etaluma Lumascope LS720 microscope with a 60X objective (Meiji MA969) (scale bar 10 μm). Significance was calculated using a mixed-effects model to analyze the data, and for multiple comparisons correction, Tukey’s test was performed. (**** *p* value < 0.0001, ** *p* value < 0.0021, * *p* value < 0.05).

**Figure A1:**
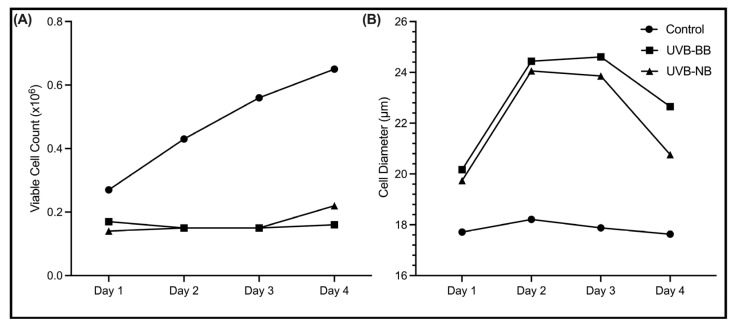
UV irradiation decreases cell proliferation and increases cell diameter. (**A**) The proliferation curve of N/TERT1 cells at 1, 2, 3, and 4 days following 6 UV irradiations with either NB-UVB or BB-UVB as compared to an unirradiated control sample. (**B**) Cell diameter of N/TERT1 or cells after 6 UV irradiations (50–70 cells/condition). The photos were taken on an Etaluma Lumascope LS720 microscope with a 60X objective (Meiji MA969) (scale bar 10 μm). Significance for N/TERT1 samples was calculated by a two-way ANOVA test and corrected for multiple comparisons using Dunnett’s test (both the day of measurement and the UV radiation were significant as compared with Day 1 and the control, respectively, *p* < 0.05).

Another robust marker of malignant transformation is nuclear enlargement which is a characteristic of cellular response to inflammation or injury [63]. N/TERT1 cells exposed to repeated irradiation of either BB-UVB or NB-UVB demonstrated an increase in nuclear size, which was similar for both types of irradiation (Figure 1B). Similarly, increased cell size is associated with decreased proliferative capacity and increased commitment to terminal differentiation [64]. Although an increase in the size of HaCaT cells was only observed at Day 2 following BB-UVB irradiation (Figure 2B), the proliferation of HaCaT keratinocytes was significantly impaired upon treatment with NB-UVB or BB-UVB (Figure 2A). In N/TERT1 cells, we observed an increased size and impaired proliferation (Figure A1). These results indicate that irradiating keratinocytes with BB-UVB or NB-UVB leads to increased recruitment of γH2AX (i.e., DSBs), nuclear enlargement, increased size, and decreased proliferation, as expected.

### 3.2. UV Irradiation Is Associated with LINE-1 Reactivation

**Figure 3:**
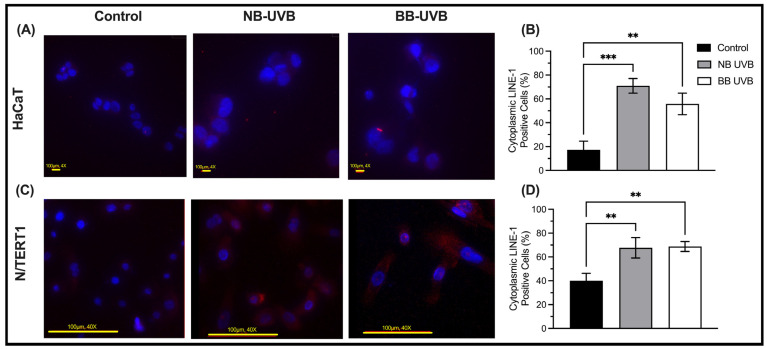
LINE-1 protein expression on HaCaT and N/TERT1 cells after 6 UV exposures with immunofluorescence. The expression of ORF1 proteins of LINE1 elements (red) as counterstained by DAPI (blue) in HaCaT (**A**,**B**) and N/TERT1 (**C**,**D**) keratinocytes as shown by immunofluorescence staining after 24 h of 6 UV irradiations with either NB-UVB or BB-UVB as compared to unirradiated control samples. The photos were taken on an Etaluma Lumascope LS720 microscope with a 60X objective (Meiji MA969) (scale bar 20 μm). Significance was calculated by a one-way ANOVA test and was corrected for multiple comparisons using Dunnett’s test. (*** *p* value < 0.0002, ** *p* value < 0.0021).

**Figure 4:**
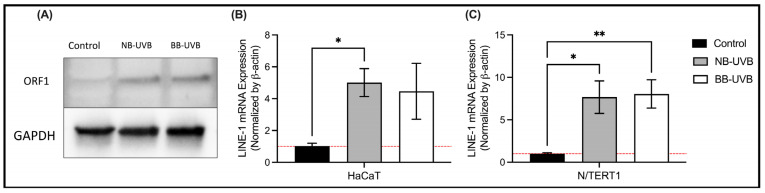
LINE-1 protein and mRNA expression in HaCaT and N/TERT1 cells after 6 UV exposures. (**A**) Measurement of ORF1 protein of LINE-1 elements using Western blotting in N/TERT1 cells. The mRNA expression of ORF2 protein of LINE-1 elements in N/TERT1 cells (*n* = 6) (**B**) and HaCaT cells (*n* = 3) (**C**) as normalized by the mRNA expression of GAPDH. Kruskal–Wallis nonparametric test was performed, and the statistical significance was corrected for multiple comparisons using Dunn’s test (** *p* value < 0.0021, * *p* value < 0.05).

**Figure 5:**
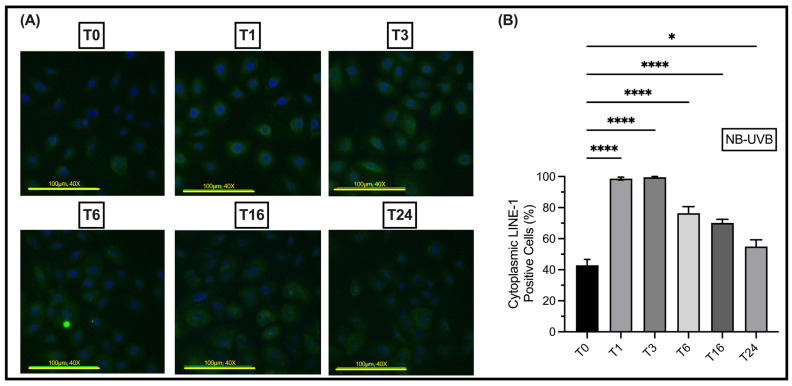
Time course of LINE-1 protein expression in N/TERT1 cells following 6 UV exposures with NB-UVB. (**A**) Immunofluorescence visualization of the expression of ORF1 protein (green) counterstained with DAPI (blue) in N/TERT1 cells (*n* = 5) after 6 irradiations with NB-UVB at different time points (0, 1, 3, 6, 16, and 24 h) (**B**) Quantification of LINE-1 expression at different time points compared with the expression immediately after NB-UVB exposure. The photos were taken on an Etaluma Lumascope LS720 microscope with a 60X objective (Meiji MA969) (scale bar 20 μm). Significance was calculated by a one-way ANOVA test corrected for multiple comparisons using Dunnett’s test. (**** *p* value < 0.0001, * *p* value < 0.05).

UV irradiation leads to the upregulation of LINE-1 ORF1p and ORF2p protein expression, and its mRNA transcripts. In N/TERT1 as well as in HaCaT cells, repeated exposure to either BB-UVB or NB-UVB resulted in increased expression of ORF1p, as visualized by immunofluorescent staining **(**Figure 3A–D). Consistent with these findings, the Western Blotting analysis showed increased expression of ORF1p in N/TERT1 cells upon repeated irradiation with either BB-UVB or NB-UVB as compared to the unirradiated control cells (Figure 4A). For additional blots of LINE-1’s protein quantification, see Figures S1 and S2. To concomitantly evaluate the activation of *LINE-1* at the mRNA level, we measured the ORF2p mRNA in N/TERT1 and HaCaT cells after repeated UV irradiations. The mRNA expression of *LINE-1* ORF2p was robustly upregulated in N/TERT1 cells with either NB-UVB or BB-UVB. However, it was heterogeneously upregulated in HaCaT cells, whereas NB-UVB irradiation led to a significant increase, and BB-UVB irradiation resulted in a trend of increasing ORF2p mRNA as compared to the unirradiated control (Figure 4B,C). Furthermore, to better understand the timeline of *LINE-1* reactivation, the expression of LINE-1 proteins in N/TERT1 cells was evaluated at different time points (1, 3, 6, 16, and 24 h) post-irradiation as compared to non-irradiated cells (0 h). Our immunofluorescence results show that the expression of LINE-1 proteins in N/TERT1 cells increases upon NB-UVB irradiation as compared to unirradiated cells starting at the 1-hour timepoint and remains high for 24 h (Figure 5A,B). The expression of LINE-1 proteins peaks between 1 and 3 h, and then gradually decreases afterwards. Therefore, our findings suggest that the irradiation of keratinocytes using BB-UVB or NB-UVB results in the reactivation of *LINE-1* elements and the increased expression of its mRNA transcripts and proteins.

### 3.3. LINE-1 Reactivation and Cellular Senescence

**Figure 6:**
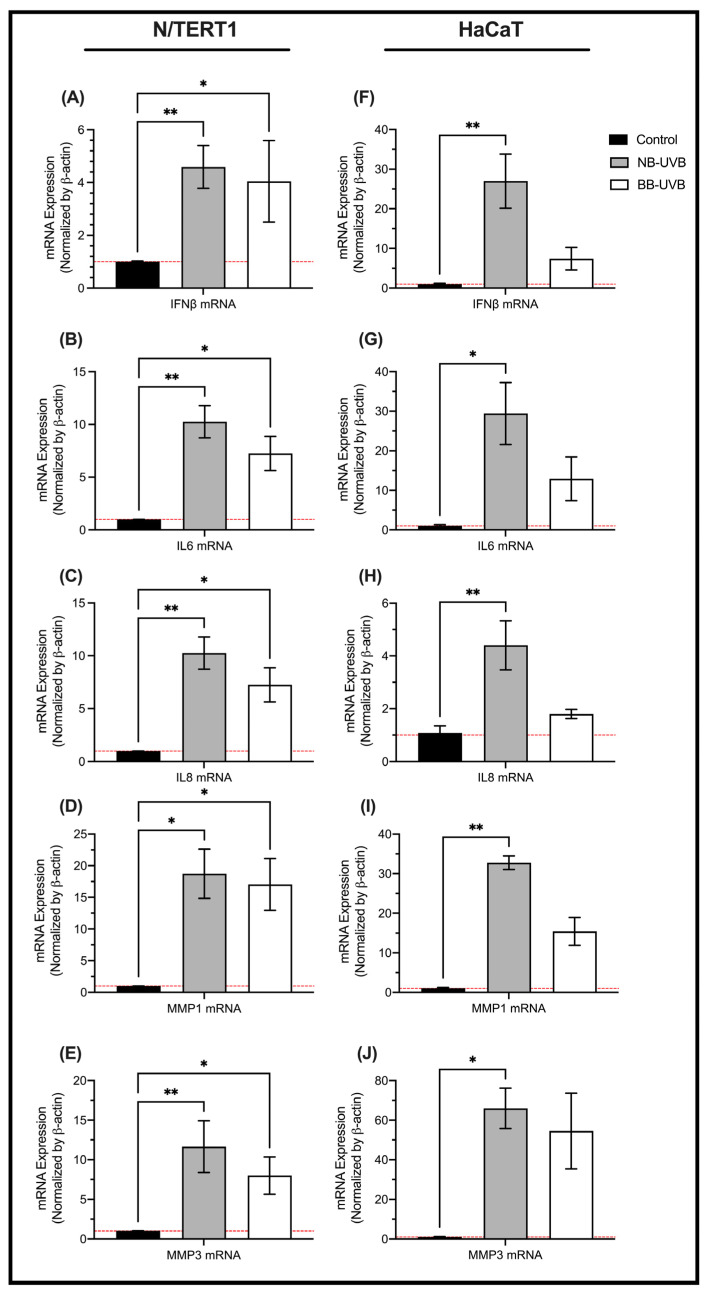
The mRNA expression of senescence markers following multiple UV exposures. The mRNA expression of senescence markers in N/TERT1 cells (*n* = 4) (**A**–**E**) and HaCaT cells (*n* = 3) (**F**–**J**) following 6 UV irradiations. Significance was calculated by the Kruskal–Wallis nonparametric test using the uncorrected Dunn’s test (** *p* value < 0.0021, * *p* value < 0.05).

To test the hypothesis of cellular senescence being associated with UV irradiation, we measured the mRNA expressions of senescence markers in N/TERT1 and HaCaT cells upon repeated UV irradiation. On one hand, the mRNA expression of senescence markers IFN-β, IL-6, IL-8, MMP1, and MMP3 was robustly upregulated upon either NB-UVB or BB-UVB repeated irradiation in N/TERT1 cells, compared to the non-irradiated controls (Figure 6A–E). However, NB-UVB irradiation had a slightly more potent effect in upregulating the mRNA expression of the senescence makers. On the other hand, the mRNA expression of the aforementioned senescence markers in HaCaT cells was heterogeneously upregulated upon repeated UVB irradiation. Whereas NB-UVB irradiation of HaCaT cells led to significant increase in the mRNA expression for all senescence markers tested as compared to the unirradiated control, BB-UVB irradiation resulted in a trend of increased expression (Figure 6F–J). Therefore, NB-UVB irradiation of HaCaT and N/TERT1 cells led to increased mRNA expression of senescence markers, whereas BB-UVB irradiation robustly upregulated the mRNA expression of senescence markers in N/TERT1 and less robustly in HaCaT cells. These results show that the UV treatment induces the upregulation of cellular senescence markers, but less robustly in p53-mutated, immortalized HaCaT cells.

### 3.4. LINE-1 Reactivation and Associated miRNAs Expression Changes

**Figure 7:**
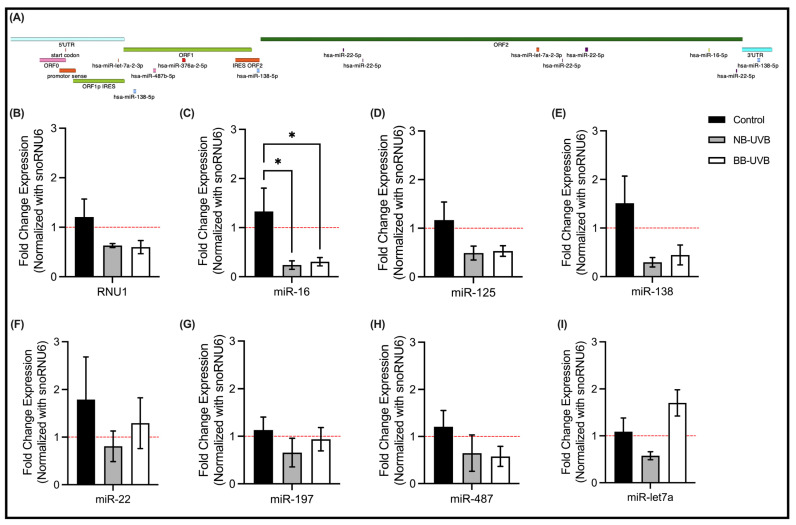
The expression of regulatory RNAs of *LINE-1* reactivation. (**A**) *LINE-1* mRNA sequence and its microRNAs binding sites. The microRNAs selected are those from the cross-search between microRNAs dysregulated upon UV irradiation and the ones that are able to target *LINE-1* mRNA. (**B**–**I**) The expression of selected miRNAs in N/TERT1 cells following 6 repeated UV exposures (*n* = 4). Significance was calculated by one-way ANOVA using Fisher’s LSD test (* *p* value < 0.05).

To investigate the RNA-based regulation of *LINE-1* reactivation, we in silico identified overlapping miRNAs by comparing datasets of miRNAs deregulated during UV irradiation [65,66] with that of miRNAs that could target *LINE-1* (using http://mirdb.org, accessed on 12 February 2023) (Figure 7A). Select shortlisted candidates including miRNAs known to be dysregulated upon UV irradiation and miRNAs that are known to target *LINE-1* mRNA based on experimental assays were then evaluated in N/TERT1 cells for their expression changes in response to UV irradiation. We specifically tested miRNAs that bind different regions in the *LINE-1* gene: *hsa-let-7a-2-3p* (*miR-let7a* afterwards) (5′UTR and IRES ORF); *hsa-miR-487b-5p* (*miR-487* afterwards), *hsa-miR-125b* (*miR-125* afterwards), *hsa-miR-197* (*miR-197* afterwards) (ORF1); *hsa-miR-138-5p* (*miR-138* afterwards) (IRES ORF2); and *hsa-miR-22-5p* (*miR-22* afterwards), *hsa-miR-16-5p* (*miR-16* afterwards) (ORF2). Additionally, we evaluated the expression of *RNU1* which was shown to be a DNA damage sensor [50].

Our results show that only *miR-16* expression was significantly decreased in N/TERT1 cells upon repeated irradiation with either of the UVB treatments compared to the unirradiated control (Figure 7C). The expression of *RNU1*, *miR-125* and *miR-138* showed a decreasing trend following UVB treatments as compared with the control (Figure 7B,D,E). In contrast, the expression of *miR-22*, *miR-197*, *miR-487* and *miR-let7a* did not change following 6 repeated UVB irradiations (Figure 7F–I).

## 4. Discussion

In this study, we demonstrate that *LINE-1* reactivation occurs at a significant rate in response to UVB exposure, which triggers genomic instability/DNA damage and is associated with senescence/aging in normal (N/TERT1) and premalignant/p53-mutated (HaCaT) human keratinocytes. We also show that miRNAs *miR-16*, as well as, possibly, *miR-125*, *miR-138* and *RNU1* may play a role in the regulation of *LINE-1* reactivation that is associated with UVB irradiation. Importantly, based on our findings *LINE-1* activation may serve as a robust/sensitive marker of stress response to UV radiation in skin keratinocytes, which may represent an interesting biological assay to be used for testing sun filters/sunscreens. Also, *LINE-1* regulatory pathways may be potentially targeted to prevent/modulate photoaging and carcinogenesis.

This work established that both BB-UVB and NB-UVB induce DNA damage in N/TERT1 cells [57,67]. These double-stranded breaks are the direct effect of the UVB-induced dimerization of pyrimidines and formation of UV products (CPDs, 64PPs) [20,68,69,70]. Moreover, a larger nuclear size was associated with repeated UV irradiations which represents a marker of cellular injury and apoptotic transformation [63]. Additionally, a decreased proliferation of N/TERT1 and HaCaT cells was observed following repeated irradiation with either BB-UVB or NB-UVB. Although UV-induced DNA damage has been shown in previous studies [21,71], we aimed to confirm the deleterious effects of UV on our cell lines given our experimental settings.

Then, we aimed to show that UVB exposure leads to the reactivation of *LINE-1* retrotransposons in normal keratinocytes. We approached this aim using three methods: immunofluorescence, Western blotting and RT-qPCR. The methods we used measure the expression of ORF1 and ORF2, which are the most robust indicators of *LINE-1* expression [72]. In addition, the immunofluorescence results provided us with insight into the cellular localization of the ORF1p, and the qRT-PCR provided a more robust measurement of *LINE-1* mRNA targeting ORF2. Western blotting showed an increased expression of LINE-1 ORF1 protein following UV irradiation in N/TERT1 cells. Moreover, using immunofluorescence, we confirmed that early UV-induced LINE-1 ORF1 protein increased expression in N/TERT1 cells as early as 1 h after UV exposure. The ORF1 protein was localized in the cytoplasm and the nucleus of the cell, as expected, given the transposable activity of the protein. The mRNA expression of ORF2p was increased robustly in N/TERT1 following six UV irradiations of NB-UVB or BB-UVB. However, the increased *LINE-1* mRNA expression in HaCaT cells was only observed following NB-UVB irradiation, whereas BB-UVB only resulted in a less robust increasing trend of expression. This increasing trend, although statistically insignificant, can be indicative of a degree of *LINE-1* reactivation from a molecular point of view.

The observed difference between HaCaT and N/TERT1 cells could be explained by the immortalization process and transcriptional profile of these cell lines. The HaCaT cells are immortalized human adult trunk keratinocytes that were derived from a lesional skin in the distant periphery of a melanoma [54]. The transformation of HaCaT cells was propagated by different Ca^2+^ culture conditions and elevated temperature [54].

Importantly, HaCaT cells retain UV-indicative p53 mutations and chromosomal aberrations similar to those present in cutaneous squamous cell carcinoma cells [54,73,74]. Therefore, HaCaT cells are often referred to as premalignant and represent an early stage of skin carcinogenesis with elevated telomerase activity and stable telomere length [73,75,76]. In contrast, the N/TERT1 immortalized keratinocyte cell line is generated from human neonatal foreskin primary keratinocytes by transducing them with the human telomerase reverse transcriptase (hTERT) gene and by the spontaneous loss of the pRB/p16^INK4a^ cell cycle control mechanism [52,53]. The gene expression pattern in N/TERT1 cells is closer to normal keratinocytes when compared to that of HaCaT cells. The stress-induced premature senescence of N/TERT1 cells is triggered by nonspecific DNA damage and is not associated with telomere shortening due to the immortalization process that helps them maintain telomere length and prevent replicative senescence [77]. The telomerase activity in N/TERT1 cells protects them from stress-induced apoptosis and necrosis but not from senescence as it is not an effective DNA repair mechanism [77]. Therefore, HaCaT cells are more prone to undergo apoptosis in response to irradiation stress than N/TERT1 cells which are more resistant. Given that more N/TERT1 cells survive repeated UV irradiations, they are more likely to have increased activation of *LINE-1* elements and upregulated senescence markers as compared with HaCaT cells.

To investigate whether UV irradiation leads to cell senescence, we measured the mRNA expression of multiple senescence markers including IFN-B, IL-6, IL8, MMP1 and MMP3 [78,79] following several UV exposures using qRT-PCR. The measured markers are part of the senescent-associated secretory phenotype, which could alter the microenvironment to promote inflammation and a consequent apoptotic transformation [80]. BB-UVB and NB-UVB light led to robust increase in mRNA expression for all senescence markers in N/TERT1 cells. However, these findings differed in HaCaT keratinocytes, where only NB-UVB led to robustly increased mRNA expression of senescence markers, whereas BB-UVB resulted in an increasing trend of senescence markers’ mRNA expression. These contrasting findings between N/TERT1 and HaCaT cells can be explained by the different immortalization techniques since HaCaT cells are more prone to apoptosis in response to repeated UV irradiations as opposed to N/TERT1 cells which are more likely to undergo stress-induced senescence [73,77]. Additionally, while NB-UVB and BB-UVB light lead to a similar inflammatory response, NB-UVB was shown to cause less DNA damage and apoptosis than BB-UVB in N/TERT1 keratinocytes and HaCaT cells for a given dose of irradiation [81,82]. Additionally, studies have demonstrated that NB-UVB is better tolerated in a clinical setting and results in fewer adverse effects [12,83]. Therefore, the lack of changes in senescence markers observed in HaCaT cells when treated with BB-UVB could be due to the predilection of HaCaT cells to undergo stress-induced apoptosis instead of senescence.

Finally, we observed a significant decrease in *miR-16* expression in N/TERT1 cells following NB-UVB irradiations. *MiR-16* has been shown to play a tumor suppressor role, and its loss or downregulation was associated with senescence and tumorigenesis, such as in cutaneous-T-cell lymphoma CTCL [84,85,86]. Moreover, *miR-16* and *miR-15a* are clustered on chromosome 13 (13q14). This chromosomal region is known to be downregulated or deleted in B-cell chronic lymphocytic leukemia (B-CLL) [87]. A large proportion of B-CLL patients carry *miR-16* genetic alterations which suggests an early contribution of the *miR-16* loss to carcinogenesis [84]. *miR-16* was also shown to serve as a biomarker for melanoma progression since the reduction in its serum levels was correlated with tumor thickness, ulceration, and stage [88,89]. Given that *miR-16* is localized to *LINE-1* ORF2, the observed robust reduction in its expression is suggestive of a putative regulatory role it may have over the UV-induced reactivation of *LINE-1*.

## 5. Limitations

One limitation of this study is the use of an immortalized keratinocyte cell lines, HaCaT and N/TERT1, as opposed to primary keratinocytes. Immortalized cell lines have a higher resistance to senescence induction compared to that of primary cells, which may affect the generalizability of the findings to primary cell models. This limitation should be considered when interpreting the results and may warrant further investigation in primary cell cultures for a more comprehensive understanding of the phenomenon. Additionally, it is important to note that some experiments, such as the proliferation assay, were conducted with relatively small sample sizes, which may introduce variability and limit the statistical robustness. Moreover, variations in sample sizes between the HaCaT and N/TERT1 cell lines could potentially influence the comparative analysis; and therefore, larger sample sizes and consistent cell numbers would be beneficial for future studies.

## Data Availability

All available data are presented in the current manuscript.

## Supplementary Materials

The following supporting information can be downloaded at: https://www.mdpi.com/article/10.3390/biomedicines11113017/s1, Figure S1: Blots used for protein quantification using imageJ (v1.53k) and Western blot images (Figure 4A); Figure S2: Additional blots used for protein quantification using imageJ.

## Appendix A

**Table A1:** The primer sequences used in qPCR experiments for senescence markers and *LINE-1*.

Gene Name	Primer Sequence
ORF2	FW-TCATAAAGCAAGTCCTCAGTGACC
	RV-GGGGTGGAGAGTTCTGTAGATGTC
IFN-B	FW-5′ACGCCGCATTGACCATCTAT-3′
	RV-5′GTCTCATTCCAGCCAGTGCT-3′
MMP1	FW-5′AGCCTTCCAACTCTGGAGTAATGT-3′
	RV-5′CCGATGATCTCCCCTGACAA-3′
MMP3	FW-5′CCCACCTTACATACAGGATTGTGA-3′
	RV-5′CCCAGACTTTCAGAGCTTTCTCA-3′
IL-6	FW-5′CACTGGCAGAAAACAACCTGAA-3′
	RV 5′ACCAGGCAAGTCTCCTCATTGA-3′
IL-8	FW-5′GTTTTTGAAGAGGGCTGAGAATTC-3′
	RV- 5′CCCTACAACAGACCCACACAATAC-3′
β-actin	FW- 5′CCAACCGCGAGAAGATGA-3′
	RV- 5′CCAGAGGCGTACAGGGATAG-3′

**Table A2:** The GeneGlobe IDs of miRCURY LNA miRNA PCR assays.

miR	miRCURY LNA miRNA Custom PCR Assays
RNU1A1	GeneGlobe ID- YP00203909 (Qiagen)
hsa-miR-16-5p	GeneGlobe ID- YP00205702 (Qiagen)
hsa-miR-125b-1-3p	GeneGlobe ID- YP00204400 (Qiagen)
hsa-miR-138-5p	GeneGlobe ID- YP00206078 (Qiagen)
hsa-miR-22-5p	GeneGlobe ID- YP00204255 (Qiagen)
hsa-miR-197-3p	GeneGlobe ID- YP00204380 (Qiagen)
hsa-miR-487b-5p	GeneGlobe ID- YP02115384 (Qiagen)
hsa-let-7a-5p	GeneGlobe ID- YP00205727 (Qiagen)
U6 snRNA	GeneGlobe ID- YP00203907 (Qiagen)
