# Supplementary material for: The Ultraviolet Irradiation of Keratinocytes Induces Ectopic Expression of LINE-1 Retrotransposon Machinery and Leads to Cellular Senescence
Source: Biomedicines. 2023 Nov 10;11(11):3017. doi: 10.3390/biomedicines11113017 (PMC10669206; doi:10.3390/biomedicines11113017)
Supplement: Supplementary file 1 [file biomedicines-11-03017-s001.zip › biomedicines-2602307-supplementary.pdf]

Figure S1: Blots used for protein quantification using imageJ and Western blot images (Figure 4A).

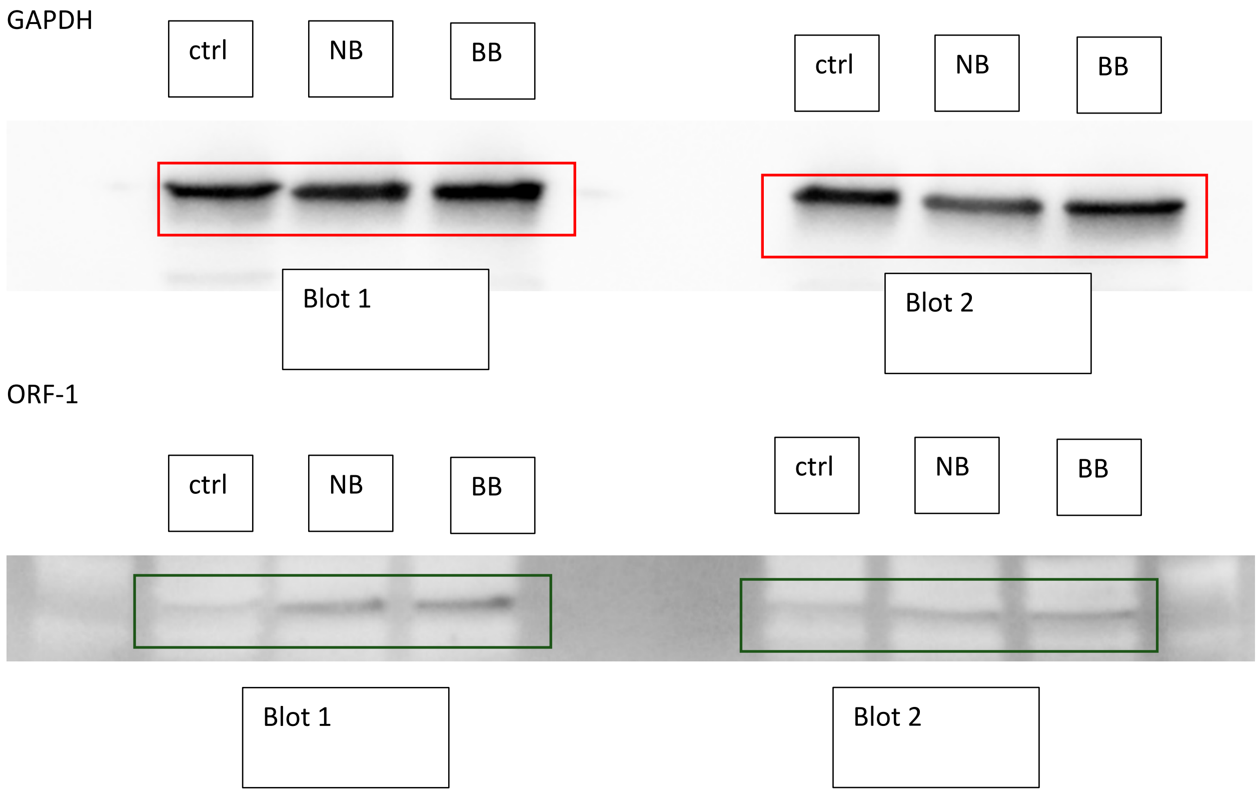

Figure S2: Additional blots used for protein quantification using imageJ.

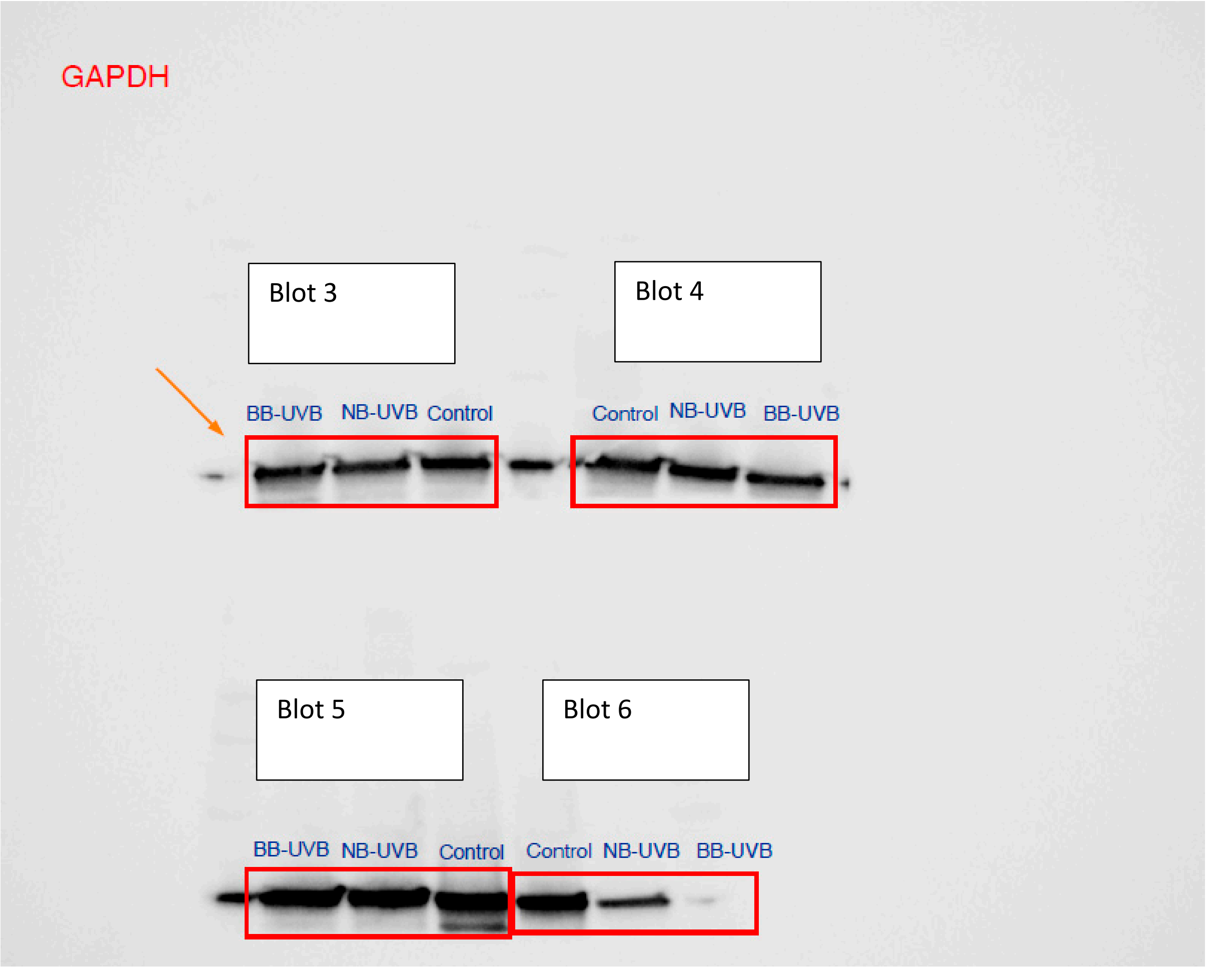

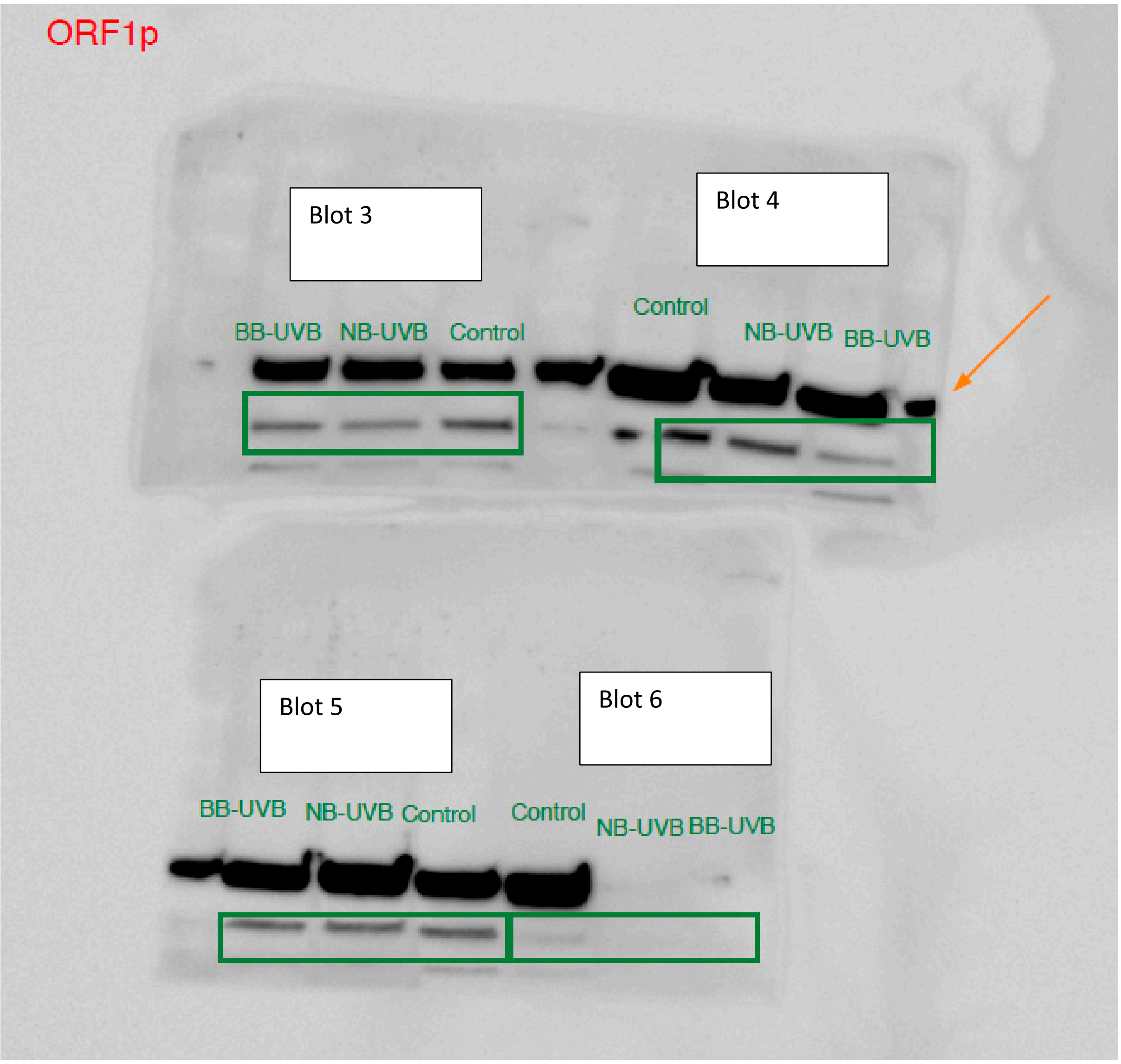
ORF1p
Blot 3
Blot 4
BB-UVB
NB-UVB
Control
Control
NB-UVB
BB-UVB
Blot 5
Blot 6
BB-UVB
NB-UVB
Control
Control
NB-UVB
BB-UVB
